# Ureteroscopy-assisted retrograde nephrostomy for a large and obstructive renal pelvic stone: a case report

**DOI:** 10.1186/s13256-015-0529-4

**Published:** 2015-02-27

**Authors:** Takashi Kawahara, Hiroki Ito, Hideyuki Terao, Hiroji Uemura, Yoshinobu Kubota, Junichi Matsuzaki

**Affiliations:** 1 Department of Urology, Ohguchi Higashi General Hospital, 2-19-1, Irie, Kanagawa-ku, Yokohama City, Kanagawa Japan; 2 Department of Urology, Yokohama City University Graduate School of Medicine, 3-9, Fukuura, Kanazawa-ku, Yokohama City, Japan

**Keywords:** PCNL, UARN, Ureteroscopy

## Abstract

**Introduction:**

We have previously described the use of ureteroscopy-assisted retrograde nephrostomy. However, reaching the target calyx with the ureteroscope is difficult in patients with obstructive renal pelvic stones.

**Case presentation:**

A 53-year-old Japanese woman was referred to our department for the treatment of a right renal stone. She was admitted to our department for percutaneous nephrolithotomy of a right renal stone located at her ureteropelvic junction. A Lawson retrograde nephrostomy puncture wire was subsequently inserted into the flexible ureteroscope, and we successfully punctured the calyx from the target spot to the skin. The nephrostomy was dilated, and the stone fragments were obtained and removed.

**Conclusions:**

We here report the case of a large and obstructive renal stone successfully treated with percutaneous nephrolithotomy using the ureteroscopy-assisted retrograde nephrostomy technique.

## Introduction

Goodwin *et al.* first reported obtaining percutaneous renal access in 1955 [1]. Subsequently, the technique of percutaneous nephrolithotomy (PCNL) was developed and has since become the standard procedure for treating large renal stones [2]. In the ureteroscopy-assisted retrograde nephrostomy (UARN) technique, it is possible to continuously visualize the area of dilation from the time of puncture to insertion of the nephron access sheath (NAS). In patients with obstructive renal pelvic stones, reaching the target calyx with a ureteroscope (URS) is difficult. In most cases, it is feasible to reach the target calyx next to large renal calculi. However, in some cases, it is not possible to reach the target calyx due to the presence of a renal stone occupying the area of the ureteropelvic junction. We here report a case of a large and obstructive renal stone successfully treated with PCNL using UARN.

## Case presentation

**Figure 1:**
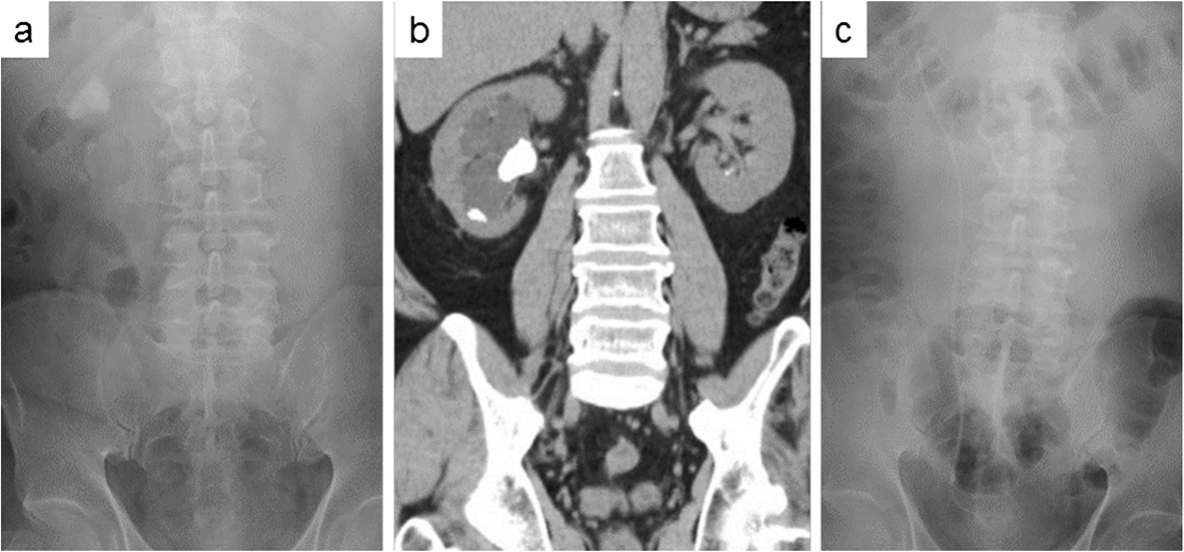
**Pre- and post- operative images. (a)** Preoperative kidney, ureter and bladder and **(b)** axial computed tomography. **(c)** Postoperative kidney, ureter and bladder.

A 53-year-old Japanese woman was referred to our department for the treatment of a right, large, obstructive renal pelvic stone and smaller lower pole calyceal stone (Figures 1a and 1b). She had no remarkable previous or family history, and the laboratory data showed no remarkable findings, except for microhematuria on a urine analysis. In April 2011, she was admitted to our department for PCNL to treat a right renal stone located at her ureteropelvic junction. In brief, under general and epidural anesthesia, she was placed in a modified-Valdivia position (Galdakao-modified Valdivia position) [3]. A flexible URS (Flex-X^2^, Karl Storz, Germany) was inserted through an inserted ureteral access sheath (Flexor® 12-Fr, 35cm, Cook Urological, USA) into her ureter. A stone occupied the area around her ureteropelvic junction, and the URS could not be advanced to the target calyx. Therefore, we first made a root using holmium:yttrium-aluminum-garnet (Ho:YAG) laser lithotripsy in order to reach the target calyx. Intracorporeal lithotripsy was subsequently performed using a Ho:YAG laser (with a 200μm fiber, 1.0J, 5Hz; VersaPulse® 30W, Lumenis Surgical, USA), and the URS was then advanced to the target spot in order to puncture it through the root.

Next, a Lawson retrograde nephrostomy puncture wire (Cook Urological) was inserted into the flexible URS, and the calyx was successfully punctured from the target spot to the skin. A 24-Fr percutaneous NAS (X-Force® nephrostomy balloon dilation catheter, BARD Medical, USA) was passed over the balloon under continuous visualization with the URS, and, following insertion of the NAS into the renal collecting system, calculus fragmentation was undertaken using a Swiss LithoClast® pneumatic lithotripter (EMS, Switzerland) through a rigid nephroscope (percutaneous nephroscope, Karl Storz, Germany). A postoperative kidney, ureter and bladder film showed no residual stones (Figure 1c). The length of the operation was 175 minutes; no major or minor complications were observed. The postoperative ureteral stent was removed 2 weeks after the procedure, and no residual stone fragments or signs of postoperative ureteral stricture were noted on computed tomography . A stone analysis showed the stone to be composed of calcium oxalate.

## Conclusions

We previously reported the application of UARN and confirmed its effectiveness during PCNL [3]. With the UARN technique, the nephrostomy can be made easily at the target puncture site under continuous visualization. The present procedure for performing UARN during PCNL has several advantages. For example, after the needle exits through the skin, no further steps are required in preparation for dilation [3-5].

In recent years, major advances have made observation of the renal pelvis easier, making it possible to perform a wide variety of intrarenal procedures using a URS [3]. Therefore, it is now easier to approach the desired renal calyx using a flexible URS than was previously possible using the fluoroscopic approach [4,6]. In our experience with UARN, the URS can be applied to continuously obtain the ideal angle.

As observed in the current case, the URS sometimes is unable to reach the renal collecting system due to the presence of a large stone at the ureteropelvic junction. Making the nephrostomy in cases of hydronephrosis is easy; however, puncturing the target calyx is important in order to free the stone in cases similar to the present case.

We here reported a case of a large and obstructive renal stone successfully treated with PCNL using UARN.

## Consent

Written informed consent was obtained from the patient for publication of this case report and any accompanying images. A copy of the written consent form is available for review by the Editor-in-Chief of this journal.

## Abbreviations

Ho:YAG: Holmium:yttrium-aluminum-garnet
NAS: Nephron access sheath
PCNL: Percutaneous nephrolithotomy
UARN: Ureteroscopy-assisted retrograde nephrostomy
URS: Ureteroscopy
NAS: Nephro access sheath
